# Mapping Nursing Telemedicine Practices: A Scoping Review of Models, Outcomes, and Professional Roles

**DOI:** 10.3390/nursrep16050161

**Published:** 2026-05-09

**Authors:** Blerina Duka, Kejda Nuhu, Fabiola Mane, Jola Çini, Armela Zylfo, Kujtime Vakeflliu, Alta Arapi

**Affiliations:** 1 Department of Medicine, Barleti University, 1000 Tirana, Albania; co.research@umb.edu.al (K.N.); koord.mjekesi-tek@umb.edu.al (F.M.); j.xharo@umb.edu.al (J.Ç.); a.zylfo@umb.edu.al (A.Z.); k.vakeflliu@umb.edu.al (K.V.); 2 Albanian Order of Nurses, 1000 Tirana, Albania; alta_arapi@yahoo.com

**Keywords:** telenursing, telemedicine, digital health, remote patient monitoring, nursing competencies, chronic disease management

## Abstract

**Background/Objectives**: The rapid expansion of telemedicine has reshaped healthcare delivery, positioning telenursing as essential for continuity of care and patient management. This scoping review maps current evidence on telecare nursing practices, examining organizational models, professional roles, and key clinical and organizational outcomes. **Methods**: The review was conducted across five international databases, following the methodological framework proposed by Arksey and O’Malley, the interpretive extension by Levac et al., and the Joanna Briggs Institute guidelines, with reporting aligned to PRISMA-ScR recommendations. The search identified 1760 records, of which 1219 remained after duplicate removal. After title and abstract screening and full-text evaluation, 25 studies met the inclusion criteria. **Results**: Telenursing was implemented across diverse clinical contexts, particularly in chronic disease management, oncology, postoperative care, and emergency settings. Evidence indicates improvements in symptom management, therapeutic adherence, quality of life, and complication reduction, suggesting positive clinical and organizational impacts. The literature highlights the need for advanced digital, communication, and relational competencies, emphasizing the importance of targeted professional training. Cross-cutting trends include enhanced continuity of care, greater patient autonomy, improved integration between hospital and community services, and reduced healthcare costs. **Conclusions**: This review provides an updated overview of telenursing applications, highlighting their adaptability across clinical settings and the expanding strategic role of nurses in digital care. The findings indicate a rapidly evolving field and emphasize the need for further research to strengthen organizational frameworks, define advanced competencies, and support the sustainable integration of telenursing into healthcare systems.

## 1. Introduction

Telemedicine has become a central driver of transformation in contemporary healthcare systems, signifying a fundamental structural shift in the organization, delivery, and patient experience of care [1]. The technological evolution of recent years, accelerated decisively during the COVID-19 pandemic, has exponentially expanded the use of digital platforms for healthcare delivery, profoundly altering traditional interaction patterns between professionals and patients [2,3]. In this scenario, the nursing profession has assumed a central role, becoming a key factor in remote care models and contributing to the development of innovative pathways capable of integrating proximity, continuity, and service sustainability [4,5]. Nursing telemedicine, defined as the provision of nursing care through digital technologies and telematic platforms, constitutes not merely a technological extension of clinical practice but a paradigmatic transformation encompassing care processes, professional roles, and modes of therapeutic interaction [6].

In recent years, international literature has documented the progressive consolidation of nursing role in telemonitoring, teleassistance, and teleconsultation [7,8]. Across various clinical contexts, nurses have become central figures in the remote management of chronic conditions, in the ongoing surveillance of vital parameters, in therapeutic education, and in the coordination of interprofessional healthcare pathways [9,10]. Their ability to integrate clinical judgment, communication skills, and digital competencies positions them as the professionals most suitable for delivering care via connected technologies, particularly where frailty, chronicity, or geographical barriers hinder access to traditional services. Recent studies, including those by Lee et al. (2022), Jiang et al. (2023) and Mahvar et al. (2025) [11,12,13], confirm that nursing interventions delivered through telemedicine can reduce rehospitalizations, improve treatment adherence, stabilize clinical parameters, and increase patient satisfaction [14].

The expansion of nursing telemedicine has been facilitated both by organizational factors and by broader systemic transformations. Digitalization has emerged as a strategic response to growing pressure on healthcare services, workforce shortages, and the need to guarantee continuity of care in aging populations with complex needs [15]. Teleassistance enables more efficient monitoring of chronic patients, optimizes nurses’ workload in community settings, and supports the creation of more sustainable models from an organizational perspective [16]. Telemedicine further strengthens person-centered care by enabling patients to receive services within their home environments and maintain greater autonomy [17]. At the same time, the relational dimension of care is supported through more frequent, timely, and sustained digital communication between nurses and patients [18].

Recent literature highlights how telemedicine is reshaping the professional role and competencies of nurses, requiring advanced competencies in digital literacy, data interpretation, autonomous clinical decision-making, and remote triage [19,20,21]. Nurses working in telemedicine increasingly operate as coordinators of care, mediators of communication processes, proactive managers of early clinical deterioration, and facilitators of patient and caregiver engagement. McConnell et al. (2023) emphasize that digitalization demands the development of new forms of operational leadership, whereby nurses become agents of innovation who support the implementation of telematic systems within healthcare organizations [22,23].

The rapid expansion of nursing roles across diverse telemedicine applications has not been accompanied by a parallel standardization of terminology and conceptual frameworks. As nursing activities have extended into multiple domains, ranging from telemonitoring to virtual consultation, different models have emerged, often described using inconsistent and overlapping terms. This variability has contributed to increasing terminological, conceptual, and methodological heterogeneity in the literature, making it challenging to compare findings and synthesize evidence across studies.

Despite this evolution, the field of nursing telemedicine still represents substantial terminological, conceptual, and methodological heterogeneity. Terms such as “telehealth nursing”, “telenursing”, “telecare”, “virtual nursing”, and “remote nursing monitoring” are often used interchangeably, contributing to conceptual overlap and complicating comparative analyses [8,14]. As highlighted by Snoswell et al. (2024), these ambiguities have hindered the development of standardized practices, resulting in heterogeneous models that vary across geographical, infrastructural, and policy contexts [14,24].

The variability in terminology is not merely semantic but reflects substantive differences in how nursing roles are conceptualized and operationalized across telemedicine models. For instance, “telenursing” is often associated with direct clinical interventions such as remote monitoring and patient education, whereas “telehealth nursing” may encompass broader coordination, case management, and interprofessional communication functions. Similarly, terms such as “remote care” or “virtual nursing” may indicate varying degrees of nurse autonomy, decision-making responsibility, and involvement in care pathways.

This conceptual variability has important implications for research and practice, as it contributes to inconsistencies in how nursing activities are measured and evaluated. As a result, generating comparable evidence on key outcomes, such as workload, job satisfaction, and professional autonomy, remains challenging.

The absence of an integrated perspective is further reflected in the tendency of many studies to focus on individual pathologies or isolated care settings, thereby failing to provide a comprehensive understanding of the nursing role across remote care models.

Although robust evidence exists on the clinical effectiveness of telemonitoring and telecare for several conditions, fewer studies examine the organizational and professional implications of nursing telemedicine, such as workload, job satisfaction, professional autonomy, process redesign, and digital skill development [5,15,25]. The available evidence suggests that digital transformation requires profound cultural change within healthcare organizations and that nurses must adopt dynamic, adaptive roles consistent with the functioning of complex and evolving systems [26].

Telemedicine also introduces new ethical and relational dimensions. Issues such as digital data management, privacy protection, quality of remote communication, construction of the therapeutic relationship through virtual interfaces, and equity of access to technologies are increasingly central [27,28]. Nurses, due to their close relational engagement with patients’ lived experiences, play a critical role in ensuring that digital care remains person-centered and that the empathic dimension of care is preserved within technologically mediated environments [29]. Evidence from Duffy et al. (2025) indicates that patients’ experiences with telehealth are closely related to the quality of communication established by nurses, particularly regarding clarity, support, and the ability to maintain therapeutic engagement despite physical distance [30].

In this dynamic scenario, there is a clear need for a rigorous and systematic scoping review to map the extent, nature, and characteristics of the existing literature on nursing telemedicine [31,32]. The purpose of this review is to explore the most recent evidence on nursing telemedicine, examine the models implemented, analyze the outcomes investigated, and understand how nursing roles have evolved in response to digital technologies [33]. In the absence of an exhaustive synthesis that integrates clinical, organizational, and professional dimensions, this paper aims to provide a comprehensive and updated overview to support the development of innovative care models, the training of nurses, and the design of health policies aligned with digital transformation [34]. At a time when technological innovation is redefining the very nature of care, understanding systematically the contribution of nurses to telemedicine is essential to ensure effective, equitable, and sustainable healthcare services.

## 2. Materials and Methods

### 2.1. Methodological Approach

This scoping review was performed in accordance with the PRISMA-ScR (Preferred Reporting Items for Systematic Reviews and Meta-Analyses extension for Scoping Reviews) guidelines, conducted following the original methodological framework proposed by Arksey and O’Malley (2005), subsequently expanded by Levac and colleagues (2010) and finally consolidated within the methodological guidelines of the Joanna Briggs Institute [35,36]. Arksey and O’Malley’s approach provided the fundamental structure of the entire process, articulating the review into five main steps that include identifying the research question, defining a broad and inclusive research strategy, selecting studies, mapping the extracted data, and narrative synthesis of the results [35,36]. In the review by Levac et al. (2010), these steps were further refined by attributing a central role to conceptual clarity, a more precise definition of the objective of the review and the need to adopt a structured and transparent decision-making process during the selection of studies [36]. This contribution has guided the entire process of this review, directing the methodological choices towards greater internal consistency and a constant commitment to the justification of the criteria used.

The most recent framework of the Joanna Briggs Institute has provided the operational reference for the concrete application of the methodology, in particular for the definition of the research strategy, the use of well-defined inclusion and exclusion criteria and the adoption of a systematic approach in the data extraction and synthesis phase. The integration of the three perspectives has made it possible to build a robust, transparent and reproducible procedure, while maintaining the flexibility necessary to explore an emerging and multidimensional field such as telenursing.

The entire review process was aligned with the indications of the PRISMA-ScR, which guided the reporting of the phases of the review, from the search to the selection of the studies, ensuring a high standard of quality and a clear description of the methodological decisions taken. This combination of methodological references has made it possible to develop a rigorous and articulated mapping of the available evidence on nursing telemedicine, ensuring solidity and interpretative depth to the review.

### 2.2. Definition of the Research Question

The definition of the research question followed the criteria of the Joanna Briggs Institute, which recommends the use of the PCC (Population, Concept, Context) approach.

This methodological approach is particularly well suited to scoping reviews that aim to explore multifactorial and rapidly evolving phenomena. The question was formulated with the aim of investigating the extent and nature of the contemporary literature regarding nursing telemedicine, what models have been described, what role nurses assume in different remote care programs and what clinical, organizational and professional outcomes emerge from the available studies. The construction of this question took place through a preliminary literature review and an iterative process, as indicated by Levac et al. (2010), in order to ensure a clear orientation and at the same time broad enough to include the variety of existing evidence [36].

### 2.3. Development of the Research Strategy

The research strategy was developed according to an articulated process, which began with a preliminary recognition of the recent literature in order to identify the most widely used terms in the field of nursing telemedicine. This first phase made it possible to define the key concepts and terminological variants necessary to build sensitive and specific queries. The construction of the search strings combined free-text terms with controlled vocabulary, including MeSH terms and discipline-specific thesauri, to ensure comprehensive coverage of the phenomenon under investigation. Terms related to telemedicine, nursing telemonitoring, telecare, digital nursing and remote care models were included. The final definition of the strategy was reached through an iterative and reflective process, characterized by the progressive verification of the adequacy of the results obtained and the modification of the terms if new evidence or new conceptual dimensions emerge.

### 2.4. Search Strategy and Strings Used

The research strategy was built through an iterative process, in line with methodological recommendations from Arksey and O’Malley (2005), Levac et al. (2010), and the Joanna Briggs Institute [35,36]. After an initial exploratory survey of the recent literature, it was possible to identify the terms most used in studies concerning nursing telemedicine. The definition of the keywords and controlled descriptors required particular attention to the terminological variability that characterizes this field, since in the international literature there are numerous expressions used to indicate similar models, such as “telehealth nursing”, “telenursing”, “remote nursing care”, “virtual nursing”, “telemonitoring” and other similar terms. The final search strings were developed through the integration of free-text terms and controlled vocabulary (such as MeSH), with iterative testing to verify the consistency and relevance of the retrieved results.

In biomedical area databases, such as PubMed/MEDLINE, the search was conducted using a combination of MeSH terms and keywords. Although the formulations vary slightly depending on the structure of the database queried, the main string used on PubMed included the combination of the MeSH term “Telemedicine” and keywords related to nursing, including “nursing”, “nurses”, “telenursing”, “telehealth nursing” and “remote nursing care”. One of the reference formulations, adapted to the MeSH syntax, integrated terms related to telemedicine with those related to the nursing profession, also including outcome indicators such as “patient outcomes” or “care outcomes” when useful for refining research. The overall structure involved the association of the main concepts by means of Boolean operators, with a central nucleus consisting of a combination similar to the following: “Telemedicine” [MeSH] AND (nursing OR nurses OR telenursing OR “telehealth nursing” OR “remote nursing care”).

In multidisciplinary databases, such as Scopus and Web of Science, search strategies were constructed using strings that incorporated free-text terms within the title, abstract, and keyword fields. In these platforms, where there are no controlled dictionaries similar to MeSH, the strategy was based on formulas including expressions such as “telemedicine”, “telehealth”, “telenursing”, “digital nursing”, “remote nursing monitoring” and “virtual nursing”, combined with terms related to the professional role (“nurse”, “nursing”, “nurse-led”). One of the main formulations involved the use of the combination “telemedicine” AND “nursing” as a basic structure, progressively expanded through the inclusion of synonyms and lexical variants in order to ensure maximum sensitivity of the research. Expressions referring to care outcomes, such as “clinical outcomes” and “patient experience”, have also been included, if useful for exploring more specific dimensions of the phenomenon.

In the CINAHL database, dedicated to the literature of nursing and health professions, a similar strategy was used, but further refined through the use of CINAHL Subject Headings, which made it possible to identify controlled terms similar to MeSH. Here, too, the research was built around the relationship between telemedicine and the nursing profession, using combinations such as “Telemedicine” AND “Nursing” and subsequently extending the string through terms such as “telehealth”, “telenursing”, “remote monitoring” and “virtual care”, with the aim of including the entire spectrum of digital interventions for nursing activities documented in the recent literature.

In PsycINFO, the research was oriented towards identifying studies that explored communicative, relational or psychological dimensions of telenursing telecare. For this reason, terms related to the remote therapeutic relationship, virtual communication and patient experience were used, integrated again with nursing keywords such as “nursing” and “nurse”. Also in this case, the main combination has kept the terms “telemedicine” and “nursing” as its core, then modulated with other expressions related to the psychological and communicative dimension of care provided remotely.

In summary, although the search strings have specific variations for each database, the conceptual framework that supports them remains consistent: all the searches have been built through the combination of a terminology block dedicated to telemedicine and a block dedicated to the nursing profession, enriched with terms that describe technologies, care models, professional roles and care outcomes. The definition of the strings was further refined through a preliminary verification phase, which made it possible to check the relevance of the results and correct any terminological anomalies.

### 2.5. Sources of Information and Database Research

The search was conducted in the most relevant international databases for biomedical and nursing literature, including PubMed/MEDLINE, Scopus, Web of Science, CINAHL and PsycINFO (Table 1). The selection of these data sources was driven by the need to capture both clinically focused scientific literature and research addressing organizational and behavioral dimensions of care. The review included only studies published within the past ten years and written in English or Italian, in order to ensure the relevance of the evidence to contemporary healthcare contexts. Search results were subsequently exported to bibliographic management software, which facilitated the removal of duplicates and the preparation of the dataset for the screening phase.

### 2.6. Study Selection Process

Study selection was performed using a two-stage screening process, in accordance with Joanna Briggs Institute (JBI) guidelines.

In the first phase, the title and abstract of each article were evaluated, with the aim of excluding clearly irrelevant studies. The articles considered potentially relevant were subsequently analyzed in their full text. The selection was made by two independent reviewers, who compared their respective judgments and resolved any discrepancies through discussion and consensus. This approach ensures methodological consistency and transparency, reducing the risk of misinterpretation. Studies that directly addressed the role of nurses in telehealth or that described models of telecare with nurse involvement were included.

The included studies generally referred to nursing professionals without consistently specifying the level of practice (e.g., registered nurses or advanced practice nurses). Articles that focused on technologies without a nursing care component, or that addressed the topic in a purely theoretical way without application references, were excluded.

### 2.7. Data Extraction Process

The data was extracted using a form developed following the guidelines of the Joanna Briggs Institute for scoping reviews. The form was designed to collect information relating to the context of the study, the characteristics of the participants, the technologies used, the telecare models described, the professional roles of nurses and the clinical, organizational and relational outcomes reported. The data extraction process was conducted iteratively and reflexively, allowing for the expansion or modification of analytical categories as new conceptual dimensions emerged during study review. This approach, consistent with the recommendations of Arksey and O’Malley (2005), enabled the capture of the dynamic and multifactorial nature of the phenomenon under investigation [35].

### 2.8. Data Analysis and Synthesis

The analysis phase was conducted through a narrative and thematic approach, aimed at identifying recurring patterns and relevant differences between the included studies. This method lends itself particularly well to complex phenomena such as nursing telemedicine, which involves multiple clinical, technological, organizational and relational dimensions. The thematic analysis made it possible to reconstruct the panorama of telecare nursing models, describing the functions performed by professionals in different contexts and identifying the outcomes most considered in the recent literature. The process was guided by an interpretative logic, with the aim of understanding how telemedicine is transforming nursing practice and what the repercussions are on health systems. The final synthesis was supplemented by a critical reflection on the gaps that emerged, with the aim of orienting future lines of research, as suggested by the methodological extensions of Levac and colleagues (2010) [36].

### 2.9. Quality Appraisal of Included Studies

Although scoping reviews do not traditionally require a formal quality assessment, a critical appraisal of the included studies was conducted to enhance the interpretability and strength of the evidence. The Joanna Briggs Institute (JBI) Critical Appraisal Tools were used, selecting the appropriate checklist according to the study design (e.g., randomized controlled trials, observational studies, and reviews).

The appraisal focused on methodological rigor, including clarity of inclusion criteria, appropriateness of study design, reliability of outcome measurement, and management of confounding factors. Overall, the included studies showed heterogeneous methodological quality, with stronger designs observed in randomized and quasi-experimental studies, while observational and descriptive studies presented more limitations, particularly in terms of sample representativeness and control of bias.

The results of the appraisal were not used to exclude studies but to support a more critical interpretation of the findings, highlighting areas where evidence is more robust and where further research is needed.

## 3. Results

### 3.1. Study Identification and Selection Process

The search across the five selected databases yielded a total of 1760 records, reflecting a heterogeneous distribution across the platforms consulted. Scopus was the most productive database, with 682 records retrieved due to its extensive coverage of multidisciplinary literature; Web of Science contributed 466 articles, highlighting a significant presence of studies related to both telemedicine and nursing care models; CINAHL returned 358 records, confirming its central role in research related to the nursing profession; PubMed added 208 relevant articles, consistent with its biomedical focus and PsycINFO produced 46 contributions, mainly related to the communicative, psychological and relational aspects of the nurse-patient digital interaction.

**Figure 1:**
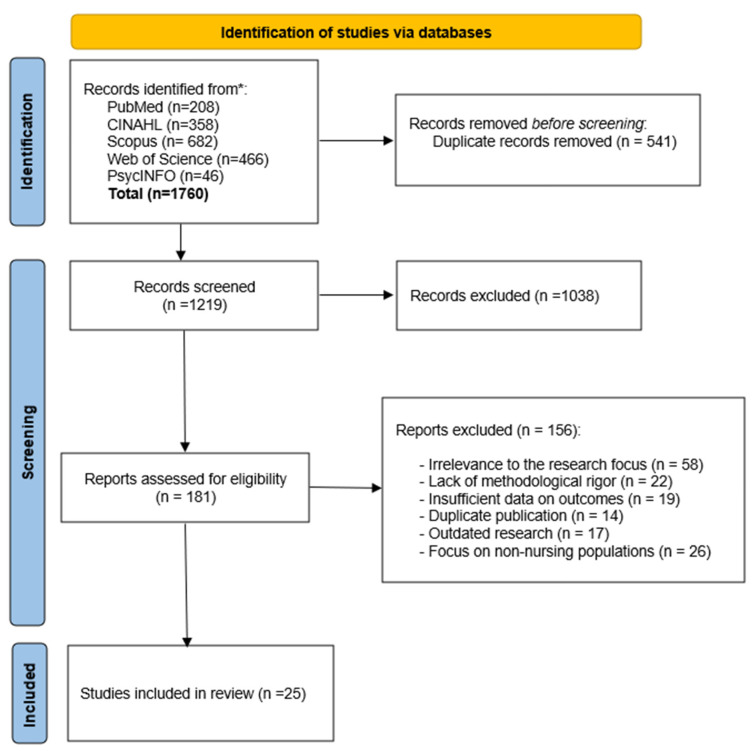
PRISMA-ScR flow diagram illustrating the selection process of the sources of evidence, including identification, screening, eligibility assessment, and final inclusion of studies. * databases queried for the scoping review.

After removing the duplicates, amounting to 541 records, the total number of unique items was 1219. These were screened for titles and abstracts. The analysis made it possible to exclude irrelevant studies, those without a significant nursing component and those that treated telemedicine exclusively from a technological point of view, without the involvement of health professionals. At the end of the full-text selection process, 25 articles were included, which constitute the final corpus of the present scoping review (Figure 1).

The distribution by clinical field, type of study and telecare model adopted highlights a considerable methodological and applicative variety of telenursing. Studies range from experimental and quasi-experimental interventions to systematic reviews, scoping reviews, study protocols, and observational studies. This heterogeneity reflects the multidimensional nature of nursing telemedicine, which can be implemented in very different clinical settings, adapting to the needs of patients and services.

### 3.2. Characteristics of the Studies Included

The studies included in the present s coping review, 25 in total, show considerable heterogeneity regarding methodological designs, clinical contexts and populations involved, reflecting the multidimensional nature of telenursing in the international literature. Table 2 provides a complete summary of the main characteristics of the 25 studies included.

The temporal distribution shows a significant concentration of publications in the last five years, with contributions that are mainly placed in the period following the COVID-19 pandemic, during which digital acceleration has promoted an increasingly extensive use of telenursing [37,38,39]. Alongside experimental and quasi-experimental studies, there are retrospective analyses, systematic reviews, feasibility studies and different research protocols, a sign of a growing scientific structuring of the field [40,41].

Geographically, the studies show a mapping spread over several continents, with a prevalence of contributions from Europe, followed by Asia, the Middle East and USA. In Europe, a significant share of works comes from Mediterranean and Central European contexts, such as Italy [42,43], Spain [44], Portugal [45] and France [46]. Asia represents a very active area in telenursing research, with several contributions from China, South Korea and Japan, especially in the oncology and postoperative fields [47,48]. The Middle East is also well represented, particularly by Iran, Turkey and Israel, with studies oriented towards post-surgical support, diabetes management and training of nursing students through advanced simulation [49,50,51]. In the Americas, the United States contributes substantially to the literature through organizational analyses and advanced digital care models [52,53], while Latin American contexts have reported more targeted applications of telemedicine in areas such as cardiology, oncology, and continuity of care [43,45].

The clinical settings covered by the studies are equally variable. The most represented sectors include oncology [47,48], diabetology [49,54], neurology and neurodegenerative diseases [40,43], surgery and postoperative follow-up [37,38], cardiology [42] and emergency [44]. There are also contributions dedicated to caregiver support, rehabilitation, pediatrics and tele-triage [55,56], outlining a rich and multidisciplinary panorama.

The technologies used vary from synchronous teleconsultation platforms to remote monitoring systems, structured phone calls, digital applications and hybrid models that combine face-to-face and remote assistance. Nursing roles described include clinical telemonitoring, therapeutic education, postoperative follow-up, symptom assessment, emotional support, specialist counseling, and coordination of complex home pathways [40,46]. This variety highlights the progressive consolidation of advanced digital skills in nursing practice, as well as the growing recognition of the role of nurses in the governance of digital care models.

Overall, the mapping of the evidence reveals a rapidly expanding sector, characterized by broad international adoption, increasingly structured organizational models, and an evolving professional nursing role. The diversity of geographical contexts, clinical settings, technologies, and methodological approaches underscores both the need for and the relevance of a scoping review capable of providing an integrated and up-to-date overview of contemporary nursing telemedicine.

**Table 2:** Summary of the studies included in the scoping review on telenursing.

N	Lead Author (Year)	Type of Study	Clinical Scope/Population	Focus of the Intervention or Review	Telenursing Modalities	Primary Domain of Outcome
1	AkbariRad et al. (2023) [48]	Narrative review	Type 2 diabetes	Effects of telenursing on disease outcomes	Remote monitoring	Metabolic clinical outcomes
2	Ali-Saleh et al. (2025) [50]	Cohort study	Nursing students	Simulation program for telenursing training	Telehealth simulation	Skills and readiness
3	Amir et al. (2025) [57]	Scoping review protocol	Various populations	Independence and economic value	Not applicable	Conceptual framework
4	Bruce et al. (2024) [51]	Retrospective cohort study	Acute care patients	Telenursing discharge program	Hospital telemonitoring	Patient and nursing experience
5	Cilia et al. (2025) [40]	Study protocol	Atypical parkinsonism	Home-based integrated care (IMPACT)	Home monitoring	Expected clinical outcomes
6	Culligan et al. (2017) [58]	Observational study	Thoracic surgery	Reducing readmissions	Telephone follow-up	Readmissions, satisfaction
7	de Souza et al. (2017) [56]	Pilot study	Catheterization patients	Telenursing intervention	Remote follow-up	Autonomy, complications
8	do Nascimento Mozer et al. (2025) [41]	Scoping review	Surgical oncology	Telenursing in cancer care	Perioperative telecare	Clinical outcomes
9	Doimo et al. (2026) [42]	Narrative review	Cardiology	Evidence on cardiology telenursing	Remote monitoring	Adherence, rehospitalization
10	Esmaeilpour-BandBoni et al. (2021) [49]	Randomized controlled trial	Older adults with diabetes	Telephone-based telenursing	Structured calls	Glycemic control
11	Gidora et al. (2019) [52]	Observational study	Triage patients	Teletriage impact	Nursing teletriage	Costs, resource use
12	Gimenez et al. (2024) [38]	Scoping review	Postoperative patients	Follow-up through telenursing	Remote monitoring	Complications, continuity
13	Hançer et al. (2023) [37]	Observational study	Postoperative patients	Outcomes during COVID-19	Remote support	Recovery, symptoms
14	Kim et al. (2023) [46]	Systematic review and meta-analysis	Colorectal cancer	Effectiveness of interventions	Remote follow-up	Clinical outcomes, QoL
15	Komariah et al. (2021) [47]	Scoping review	Lung cancer	Care delivery model	Remote assistance	Continuity of care
16	Lo Monaco et al. (2025) [53]	Umbrella review	Diabetes	Effectiveness of telenursing	Multiple models	Clinical outcomes
17	Loverre et al. (2026) [42]	Narrative review	Cardiology	Current scientific evidence	Teleassistance	Clinical and organizational outcomes
18	Mancini et al. (2020) [43]	Interventional study	Parkinson’s disease	Personalized care management	Integrated monitoring	Symptom management
19	Meunier-Sham et al. (2019) [45]	Descriptive study	Forensic nursing	TeleSANE implementation	Remote examination	Care model quality
20	Rahmiati et al. (2025) [39]	Scoping review	Various settings	Quality and cost impact	Mixed modalities	Savings, quality
21	Sarik et al. (2022) [54]	Preliminary interventional study	NICU infants and caregivers	Transition to home	Telehealth support	Caregiver stress
22	Sebastià et al. (2024) [59]	Systematic review	Emergencies and disasters	Role of telenursing	Teletriage	Safety, organizational outcomes
23	Shahrokhi et al. (2018) [55]	Interventional study	Head trauma caregivers	Post-discharge care	Remote counseling	Care quality
24	Smith et al. (2018) [60]	Educational study	Nursing students	Teaching telehealth competencies	Simulation	Professional skills
25	Toffoletto et al. (2020) [44]	Integrative review	Latin America and Caribbean	Telenursing in care and management	Multiple applications	Models, barriers

### 3.3. Methodological Quality of Included Studies

A methodological quality appraisal of the included studies was conducted using the Joanna Briggs Institute (JBI) Critical Appraisal Tools, selecting the most appropriate checklist according to each study design. The results of this appraisal are presented in Table 3.

The analysis revealed a heterogeneous level of methodological quality across the included studies. Higher levels of rigor were observed in randomized controlled trials and secondary research, including systematic reviews, meta-analyses, and umbrella reviews, which were generally characterized by structured methodologies, clearly defined inclusion criteria, and, in some cases, formal risk of bias assessment.

In contrast, observational, quasi-experimental, and descriptive studies demonstrated moderate methodological quality, often limited by the absence of randomization, potential selection bias, and variability in outcome measurement. Case studies and implementation reports provided valuable insights into real-world applications of telenursing but were characterized by lower methodological robustness.

Narrative reviews and study protocols were not formally appraised for methodological quality, as they did not provide primary outcome data or systematic synthesis processes.

Overall, the appraisal highlights that, despite the growing volume of literature on telenursing, the strength of evidence remains variable. These findings support the need for more rigorous experimental and longitudinal studies to strengthen the evidence base and enhance the generalizability of results in this field.

### 3.4. Applications of Telenursing in Chronic Diseases

The analysis of the twenty-five studies included shows that chronic conditions represent the prevailing area of application of telenursing. Evidence regarding diabetes, heart disease and Parkinson’s disease converges in documenting relevant benefits in terms of symptom management, therapeutic adherence and improvement of clinical outcomes. Interventions dedicated to the management of diabetes indicate an improvement in metabolic parameters, a greater capacity for self-management and a reduction in complications associated with the disease. Similarly, studies focused on Parkinson’s show that telenursing facilitates continuous monitoring of symptoms, supporting personalized care pathways that actively involve both patients and caregivers. In cardiovascular diseases, telenursing is used to support home management, provide personalized information and ensure constant follow-up, improving clinical stability and continuity of care. Overall, this group of studies suggests that telenursing in chronic diseases represents an effective model to support autonomy, reduce complications and promote a more stable control of clinical conditions in the long term.

### 3.5. Telenursing in Oncology Pathways

As our results demonstrated, a substantial portion of the literature focuses on oncology pathways, where telenursing provides valuable support for symptom management, monitoring, and patient education. Systematic reviews and primary studies highlight how telecare plays a central role in pre- and postoperative management, symptom control, and informational and emotional support to patients. This care modality is particularly useful in complex oncological pathways, such as those dedicated to colorectal cancer or lung cancer, in which the intensive and prolonged nature of treatment requires continuous monitoring and constant dialog between patients and professionals. Evidence shows that telenursing helps improve quality of life, reduces response times in the event of complications and promotes greater adherence to treatments. In addition, the possibility of providing remote support allows for effective integration of the care pathway, especially in periods when face-to-face access may be difficult or not strictly necessary.

### 3.6. Telecare Nursing in the Postoperative Period

Consistent with the findings reported in the results, telenursing interventions in the postoperative phase enable early detection of complications and improved continuity of care. Telecare nursing is a particularly important element in the management of the postoperative period. The included studies show that, through remote telephone or digital follow-up, it is possible to reduce complications, improve adherence to post-surgical indications and facilitate the transition from hospital to home. Remote monitoring allows nurses to detect signs of distress early, provide timely clarification of prescriptions and intervene in a targeted manner in the event of emerging symptoms. Evidence from pilot interventions also shows that structured telenursing follow-up can support patient autonomy and reduce complications in home-based care pathways [60]. This approach results in a decrease in hospital readmissions and an improvement in the overall experience of patients and their families. The literature also suggests that such interventions strengthen the sense of continuity of care, offering patients a reassuring professional presence even outside the hospital environment.

### 3.7. Telenursing in Emergency Contexts and Disasters

The results of this review also highlight the strategic role of telenursing in emergency and disaster contexts. A significant part of the studies analyzed focuses on the use of telenursing in emergency contexts, including natural disaster scenarios and the most critical phases of the COVID-19 pandemic. Evidence shows that telenursing has played a crucial role in triage, resource management, decision support and continuity of care under conditions of extreme pressure on health systems. The speed with which services were reorganized during the pandemic highlighted the flexibility of telenursing and its ability to maintain effective care even in the absence of direct physical contact. The studies describe how remote support has made it possible to manage the needs of large populations, monitor isolated patients, provide clinical indications and guide care decisions in a timely manner, contributing decisively to the resilience of the health system.

### 3.8. Digital Skills and Nursing Education

As identified in the included studies, the development of digital and relational competencies represents a key requirement for effective telenursing practice. A significant part of the literature examines the skills needed to work in telenursing and the role of training. Studies involving nursing students and professionals in training show that telecare requires advanced digital skills, specific communication skills and a conscious use of technological tools.

Telenursing should not be interpreted as a departure from traditional nursing practice, but rather as an evolution of core nursing competencies within a technologically mediated environment. Fundamental skills such as clinical assessment, patient education, and care coordination remain central, although they are adapted to remote and digital contexts. At the same time, telenursing requires the development of additional competencies, including digital communication, remote clinical reasoning, and the ability to interpret patient-generated data.

Structured simulations are particularly effective in preparing students, strengthening the ability to use digital platforms, conduct remote assessments, interact with the patient through technological tools and manage complex clinical situations remotely [57]. Evidence suggests that integrating telenursing training into university curricula and professional development programs represents a strategic step to consolidate this care model in daily practice.

### 3.9. Emerging Cross-Cutting Trends

The integration of findings across the included studies reveals several convergent trends. First, telenursing emerges as an effective means of strengthening continuity of care, particularly through remote follow-up models that enhance coordination between hospital-based services and community settings. Second, multiple studies underscore the potential of telenursing to reduce healthcare costs and promote the efficient use of resources, primarily through enhanced patient monitoring and a reduction in hospital readmissions. Another recurring theme concerns the enhancement of patient and caregiver autonomy, which is reflected in improved disease management within the home setting and greater active participation in clinical decision-making processes. Emerging research protocols are also beginning to explore the relationship between telenursing, patient independence, and economic sustainability [61]. Overall, the evidence indicates that telenursing constitutes a flexible and adaptable model of care, capable of addressing diverse clinical needs while ensuring quality, safety, and continuity of care. The variety of the included studies enable the delineation of a comprehensive and detailed overview of contemporary nursing telemedicine applications, offering an up-to-date and articulated representation of current practices across healthcare settings.

The economic and organizational implications of telenursing remain a critical but often underexplored area. Despite the growing adoption of telehealth, many nursing activities within these models are not directly reimbursable, particularly in systems where billing structures prioritize physician-led services.

This lack of financial recognition may represent a barrier to the sustainable implementation of telenursing, despite its contribution to improved patient outcomes and continuity of care. Therefore, greater attention should be paid to the development of policies that recognize and support the economic value of nursing activities in telehealth contexts.

In addition, organizational strategies and targeted training programs are essential to support nurses in adapting to these evolving models of care, ensuring both professional sustainability and system-level effectiveness.

### 3.10. Integration of Findings Across Organizational Models, Roles, and Outcomes

Beyond the descriptive categorization by clinical area, a cross-cutting analysis of the included studies reveals that telenursing practices can be systematically interpreted through three interconnected dimensions: organizational models, professional roles, and clinical and organizational outcomes.

From an organizational perspective, telenursing interventions are predominantly structured within three main models: remote monitoring programs, teleconsultation-based follow-up, and hybrid care pathways integrating in-person and digital services. These models are consistently designed to enhance continuity of care, reduce fragmentation between hospital and community settings, and optimize resource allocation. Across different clinical contexts, telemonitoring emerges as the most frequently adopted model, particularly for chronic disease management, while teleconsultation plays a key role in oncology and postoperative care pathways.

In terms of professional roles, nurses are not limited to task-oriented functions but assume complex and evolving responsibilities. The evidence highlights four main role configurations: clinical monitoring and early detection of deterioration, patient education and self-management support, coordination of care pathways, and relational mediation within digital environments. These roles reflect a shift toward more autonomous and decision-oriented nursing practice, requiring advanced competencies in digital literacy, remote clinical reasoning, and communication.

Regarding outcomes, the literature demonstrates consistent improvements not only in clinical indicators, such as symptom control, treatment adherence, and complication reduction, but also in organizational outcomes. These include reduced hospital readmissions, improved efficiency of care delivery, enhanced patient satisfaction, and better integration between healthcare services. Importantly, these outcomes appear to be interdependent, suggesting that the effectiveness of telenursing lies in its ability to simultaneously impact clinical and system-level performance.

This integrated interpretation allows for a more comprehensive understanding of telenursing beyond disease-specific applications, positioning it as a multidimensional model of care that operates across clinical, organizational, and professional domains.

## 4. Discussion

This scoping review offers an updated and articulated overview of contemporary applications of nursing telemedicine. As demonstrated by the results of this review, telenursing emerges as a dynamic and adaptable care model capable of responding to complex clinical needs across different healthcare settings. The results show that telenursing is establishing itself not only as a tool for continuity of care, but as a real structural component of care models oriented towards chronicity, hospital-home transition, oncology, postoperative management and emergency contexts.

As highlighted in the results section, one of the most relevant aspects concerns the growing role of telenursing in the management of chronic diseases. The most recent studies indicate that telenursing can facilitate a significant improvement in self-management in patients with diabetes and other chronic diseases, contributing to the control of clinical parameters and reducing the need for repeated access to health services [47,54]. Evidence shows that remote follow-up, structured through telephone contacts or digital platforms, promotes greater therapeutic adherence and more active patient participation in the treatment pathway. These results are in line with the evolution of Chronic Care Management models, which emphasize the role of the nurse as a facilitator of continuity and integration of services.

Chronic disease management represents a well-established area of nursing practice; however, the use of digital tools to support patient self-management, such as in diabetes care, introduces new dimensions that require both technological competencies and enhanced patient engagement strategies.

A second area of growing interest concerns the application of telenursing in oncology pathways. The included studies show that telenursing represents a valuable support for symptom management, monitoring of side effects of treatments and psychological support of patients undergoing oncological surgery or complex treatments [41,47]. Telemedicine allows cancer patients to maintain continuous contact with professionals, reducing anxiety, uncertainty and the risk of complications, especially at critical moments in the therapeutic pathway. These results confirm what has already been observed in the international literature, which describes telenursing as a key element in supporting complex and emotionally demanding care pathways [58].

Telenursing also plays a central role in the postoperative period. The studies analyzed show that telenursing allows for more timely management of symptoms, rapid identification of complications and better adherence to post-surgical recommendations, reducing in some cases the rate of hospital readmissions [37,59]. The adoption of telemedicine in the hospital-home transition phases has proven to be particularly effective during the COVID-19 pandemic, a period during which telecare emerged as a fundamental resource for ensuring continuity, safety, and relational proximity in care. Evidence confirms that nursing telemedicine can improve the perceived quality of postoperative care, providing support to both patients and caregivers.

Another relevant result concerns the use of telenursing in emergency and disaster contexts. The systematic reviews in the sample show how telecare has been used as a strategic tool in emergency contexts, improving triage, clinical decision support and resource management during critical situations [48]. Under these conditions, nursing telemedicine makes it possible to optimize the distribution of staff, ensure continuous communication and support clinical safety even in the absence of direct physical presence.

A further significant contribution emerges from the studies dedicated to nursing education and digital skills. Simulation-based work shows that preparing for telenursing requires a complex set of skills that include the effective use of digital technologies, remote therapeutic communication, the ability to clinically assess electronically mediated and the management of relational dynamics in virtual environments [51]. These results confirm the urgency of integrating digital training into nursing curricula, promoting professionalism capable of operating in increasingly connected and digitized care contexts.

Overall, the summary of the data shows how telenursing contributes to improving the quality of care in multiple contexts. Evidence suggests that nursing telemedicine is not just a technological tool but is configured as a true organizational model that enhances the role of the nurse as a coordinator of care, promoter of patient empowerment and facilitator of continuity of care. Telemedicine applications emerge as cost-effective and clinically effective solutions, especially in care-intensive services and longitudinal care pathways [39] (Table 4).

The findings of this study indicate that telenursing has evolved beyond an emergency or ancillary intervention, emerging instead as a mature and consolidated model of care with a substantial impact on the quality, safety, and continuity of healthcare delivery. Evidence suggests that the structural integration of nursing telemedicine into healthcare systems can contribute to greater efficiency, reduced pressure on hospital facilities, and a substantial improvement in the patient experience. In this perspective, telenursing is one of the most promising components to support the digital transformation of healthcare, promoting models that are closer to patients’ needs, more sustainable and more oriented towards the complexity of contemporary care pathways.

While these findings highlight the potential benefits of telenursing across different clinical contexts, it is essential to adopt a critical perspective to fully understand its limitations and challenges.

While the findings of this review highlight several positive outcomes associated with telenursing, it is important to adopt a critical perspective. The available literature may be subject to publication bias, with a tendency to report successful implementations while underreporting challenges and negative outcomes.

Several critical issues should be considered. First, the use of digital technologies may exacerbate inequalities in access to care, particularly among older adults, socioeconomically disadvantaged populations, or individuals with limited digital literacy. Second, telenursing may introduce new forms of workload and cognitive burden for nurses, particularly in managing continuous streams of digital data and remote patient monitoring.

Additionally, limitations in remote clinical assessment may affect the accuracy of decision-making in certain contexts, while the absence of physical presence may impact the therapeutic relationship and the holistic dimension of care. Concerns related to data privacy and cybersecurity also represent significant challenges that require careful management.

These considerations highlight the need for a balanced and critical approach to the implementation of telenursing, ensuring that technological innovation is aligned with patient-centered and equitable care principles.

### 4.1. Limitations of the Scoping Review

An important limitation to consider is the relatively limited number of eligible studies identified, despite the growing relevance of telenursing in clinical practice.

An important finding of this review concerns the relatively limited number of eligible studies identified, despite the growing relevance of telenursing in clinical practice. This apparent discrepancy may reflect the rapid implementation of telemedicine interventions, particularly during the COVID-19 pandemic, which has not yet been matched by a corresponding development of robust and standardized research.

Furthermore, the heterogeneity in terminology and methodological approaches may have contributed to difficulties in identifying and comparing studies, potentially leading to an underrepresentation of existing evidence. This gap highlights the need for more systematic and coordinated research efforts in the field.

Although this scoping review offers a complete and systematic mapping of the available evidence, some limitations must be considered in the interpretation of the results. The primary limitation relates to the methodological heterogeneity of the included studies, which encompassed randomized controlled trials, observational studies, systematic reviews, scoping reviews, and study protocols. This variability makes it difficult to make direct comparisons and prevents a uniform assessment of the methodological quality of interventions. However, this heterogeneity reflects the emergent and multidimensional nature of telenursing and represents an intrinsic feature of scoping reviews, which aim to explore the breadth and nature of the evidence rather than evaluate its effectiveness.

Further limitations concern the predominance of studies conducted in specific contexts, such as oncology, diabetes or chronic diseases, which are overrepresented compared to other emerging clinical areas. This may limit the generalizability of the findings to other less studied clinical conditions. Moreover, the majority of studies adopt a predominantly clinical perspective, whereas research addressing the ethical, sociocultural, organizational, and interprofessional dimensions of telecare nursing remain comparatively underrepresented.

Another limitation concerns the fact that many practices have adopted technologies or digital platforms specific to the local health context. This technological variability makes the comparative evaluation of intervention models complex and limits the possibility of fully generalizing the organizational implications of the results. Finally, not all studies provide detailed data on the long-term outcomes of telenursing, an aspect that deserves further study in future research.

Despite these limitations, this scoping review offers a broad and up-to-date view of the literature, identifying established areas and fields of development that represent important opportunities for nursing research and practice.

### 4.2. Implications for Nursing Practice

The evidence that emerged from this scoping review suggests that telenursing is taking on an increasingly important role in contemporary healthcare systems, contributing to the transformation of care models in the direction of greater continuity, proximity and personalization. The literature shows how telenursing is able to enhance patients’ self-management, support complex decision-making processes and improve the care experience, particularly in longitudinal and high-intensity care pathways. The nurse is increasingly appearing as a digital mediator of the therapeutic relationship, not only as a provider of clinical interventions, but as a coordinator of care, educator, facilitator and stable point of reference in care transition pathways.

The practical implications concern first of all the rethinking of professional skills. The studies analyzed indicate the need to integrate advanced digital skills, remote communication skills and forms of clinical assessment mediated by technological tools into nursing curricula. Nursing training will have to be oriented towards the construction of a professional profile capable of operating in hybrid healthcare ecosystems, in which the relationship between physical presence and digital interaction is increasingly flexible and interdependent [51].

From an organizational point of view, the integration of telemedicine into care services requires a rethinking of processes, professional responsibilities and governance models. Studies show that effective telenursing programs are based on a clear structuring of nursing activities, multidisciplinary coordination, and digital platforms that allow for timely clinical assessment and continuity of information across the entire care pathway [52]. The systemic adoption of telenursing could help reduce pressure on hospitals, improve accessibility to services and foster more equitable and sustainable models of care.

A final element concerns the economic impact and sustainability of teleassistance models. Cost-oriented studies show that telenursing can reduce avoidable hospitalizations, optimize the use of resources, and produce significant savings for health systems, especially in chronic settings and in geographic areas with limited access to specialized services [39]. These results support the need to invest in nursing telemedicine as a strategic component to address the growing demand for care, staffing shortages and the epidemiological complexity of the populations being treated.

In addition to the need for a general reorientation of training programs, the findings of this review suggest the importance of integrating specific competencies into nursing education and professional development pathways. These include the ability to establish therapeutic relationships through digital interfaces (“webside manner”), ensuring effective communication and patient engagement in virtual settings.

Furthermore, nurses should be trained in conducting structured remote clinical assessments, including symptom evaluation, risk identification, and decision-making processes for care escalation. Competencies in digital data management, privacy protection, and cybersecurity are also essential, given the increasing use of electronic health platforms and telemonitoring systems.

Another critical area concerns the development of clinical judgment in virtual environments, particularly in triaging patients and determining the appropriate level of care. These competencies are fundamental to ensuring patient safety, continuity of care, and the effective integration of telenursing into healthcare systems. These competencies should be systematically integrated into undergraduate and postgraduate nursing curricula, as well as continuing professional development programs, to ensure the safe, effective, and sustainable implementation of telenursing within healthcare systems.

### 4.3. Future Research Directions

Despite the growing body of literature on telenursing, several important gaps remain that warrant further investigation. Future research should aim to provide more robust and comparable evidence on the long-term impact of telenursing on both patient and workforce outcomes.

Key research questions emerge from this review. First, what is the long-term impact of telenursing on nurse workload, burnout, and job satisfaction? Second, which specific telenursing models are most effective and cost-efficient in managing chronic conditions and multimorbidity, particularly in aging populations? Third, how can telenursing interventions be designed to ensure equitable access to care and reduce disparities associated with the digital divide?

Further studies are needed to explore the organizational and leadership factors that facilitate the successful implementation of telenursing within healthcare systems. Addressing these questions will be essential to support evidence-based decision-making and to guide the sustainable integration of telenursing into clinical practice.

## 5. Conclusions

Nursing telemedicine emerges as one of the most significant innovations in the digital transformation of contemporary healthcare systems. The results of this scoping review show that telenursing represents a consolidated, effective and sustainable care model, capable of improving the continuity, quality and safety of care through digital solutions integrated into traditional clinical pathways. Telenursing has demonstrated particular effectiveness in the management of chronic conditions, oncological care pathways, the postoperative period, and emergency contexts, supporting the complexity of care delivery while contributing to a reduction in pressure on healthcare services.

The literature suggests that nursing telemedicine plays a critical role in strengthening patient autonomy, facilitating hospital-to-home transition, supporting self-management, and improving patient and caregiver engagement. At the same time, teleassistance requires a redefinition of professional skills and training models, highlighting the need to structurally integrate digital preparation into nursing education pathways.

The digital transformation of healthcare, accelerated by the pandemic and demographic and epidemiological change, represents an opportunity to rethink the role of the nurse and to develop more resilient, integrated and future-oriented care models. Telenursing, in its ability to combine relational proximity and technological distance, embodies this evolution and represents a strategic resource for facing the challenges of complexity and sustainability in health services.

This scoping review provides a solid basis for developing further research, deepening the long-term outcomes of telenursing, exploring the integration of digital skills in nursing education, and evaluating the effect of telecare models in different organizational contexts. In a constantly changing healthcare landscape, nursing telemedicine is an indispensable component, destined to profoundly influence the future of the nursing profession and care itself.

While telenursing represents a promising and evolving model of care, the findings of this review should be interpreted with caution. The relatively limited number of available studies and the predominance of positive findings suggest the need for a more critical and balanced evaluation of its real impact.

Telenursing should therefore be considered not as a universally optimal solution, but as a complementary approach that requires contextual adaptation, continuous evaluation, and integration within broader healthcare systems. Future research should aim to address existing gaps and provide more robust evidence to support its sustainable implementation.

## Figures and Tables

**Table 1:** Search strings used in different databases.

Database	Search String Used	Record
PubMed	(“Telemedicine”[MeSH Terms] OR “Telemedicine”[Title/Abstract] OR “Telehealth”[Title/Abstract] OR “Telenursing”[Title/Abstract] OR “Remote Consultation”[MeSH Terms]) AND (“Nursing”[MeSH Terms] OR “Nurse*”[Title/Abstract] OR “Nursing Care”[Title/Abstract] OR “Telehealth Nursing”[Title/Abstract] OR “Virtual Nursing”[Title/Abstract]) AND (“Patient Outcomes”[Title/Abstract] OR “Care Outcomes”[Title/Abstract] OR “Clinical Outcomes”[Title/Abstract] OR “Professional Role”[Title/Abstract])	208
Scopus	(TITLE-ABS-KEY(“telemedicine”) OR TITLE-ABS-KEY(“telehealth”) OR TITLE-ABS-KEY(“telenursing”) OR TITLE-ABS-KEY(“virtual nursing”) OR TITLE-ABS-KEY(“remote nursing care”)) AND (TITLE-ABS-KEY(“nurse”) OR TITLE-ABS-KEY(“nursing”) OR TITLE-ABS-KEY(“nurse-led”) OR TITLE-ABS-KEY(“nursing role”)) AND (TITLE-ABS-KEY(“patient outcome”) OR TITLE-ABS-KEY(“clinical outcome”) OR TITLE-ABS-KEY(“care model”))	682
Web of Science	TS = (“telemedicine” OR “telehealth” OR “telenursing” OR “remote nursing” OR “virtual nursing”) AND TS = (“nursing” OR “nurse*” OR “nurse-led” OR “nursing care”) AND TS = (“patient outcomes” OR “clinical outcomes” OR “care delivery” OR “professional role”)	466
CINAHL (EBSCO)	(“Telemedicine” OR “Telehealth” OR “Telenursing” OR “Remote Monitoring” OR “Virtual Nursing”) AND (“Nursing” OR “Nurse*” OR “Nursing Care” OR “Nurse-Patient Relations”) AND (“Patient Outcomes” OR “Clinical Outcomes” OR “Care Models” OR “Professional Practice”)	358
PsycINFO	(AB(“telemedicine”) OR AB(“telehealth”) OR AB(“remote care”) OR AB(“virtual nursing”)) AND (AB(“nursing”) OR AB(“nurse”) OR AB(“nurse-led”)) AND (AB(“communication”) OR AB(“patient experience”) OR AB(“therapeutic relationship”))	46

**Table 3:** Methodological quality appraisal of included studies using JBI Critical Appraisal Tools.

N	Study	Design	JBI Tool	Appraisal
1	AkbariRad et al. (2023) [48]	Narrative review	Not applicable	Low
2	Ali-Saleh et al. (2025) [50]	Cohort study	JBI Cohort Checklist	Moderate
3	Amir et al. (2025) [57]	Scoping review protocol	Not applicable	Not appraisable
4	Bruce et al. (2024) [51]	Retrospective cohort	JBI Cohort Checklist	Moderate
5	Cilia et al. (2025) [40]	Study protocol (RCT planned)	Not applicable	Not appraisable
6	Culligan et al. (2017) [58]	Abstract/clinical trial (ongoing)	Not applicable	Low
7	de Souza et al. (2017) [56]	Pilot quasi-experimental	JBI Quasi-experimental	Moderate
8	do Nascimento Mozer et al. (2025) [41]	Scoping review	JBI SR Checklist	Moderate
9	Doimo et al. (2026) [42]	Narrative review	Not applicable	Low
10	Esmaeilpour-BandBoni et al. (2021) [49]	Randomized controlled trial	JBI RCT Checklist	High
11	Gidora et al. (2019) [52]	Scoping review	JBI SR Checklist	Moderate
12	Gimenez et al. (2024) [38]	Scoping review	JBI SR Checklist	Moderate
13	Hançer et al. (2022) [37]	Interventional controlled study	JBI Quasi-experimental	Moderate–High
14	Kim et al. (2023) [46]	Systematic review + meta-analysis	JBI SR Checklist	High
15	Komariah et al. (2021) [47]	Scoping review	JBI SR Checklist	Moderate
16	Lo Monaco et al. (2025) [53]	Umbrella review	JBI SR Checklist	High
17	Loverre et al. (2026) [42]	Narrative review	Not applicable	Low
18	Mancini et al. (2020) [43]	Case study/pilot intervention	JBI Case report	Low–Moderate
19	Meunier-Sham et al. (2019) [45]	Descriptive implementation	JBI Case series	Low–Moderate
20	Rahmiati et al. (2025) [39]	Scoping review	JBI SR Checklist	Moderate
21	Sarik et al. (2022) [54]	Quality improvement study	JBI Quasi-experimental	Moderate
22	Sebastià et al. (2024) [59]	Systematic review	JBI SR Checklist	Moderate–High
23	Shahrokhi et al. (2018) [55]	Interventional	JBI Quasi-experimental	Moderate
24	Smith et al. (2018) [60]	Educational quasi-experimental	JBI Quasi-experimental	Moderate
25	Toffoletto et al. (2020) [44]	Integrative review	JBI SR Checklist	Moderate

**Table 4:** Emerging themes identified through qualitative and thematic analysis of the 25 studies included.

Emerging Theme	Discursive Description
**Management of chronicity and support for self-management**	Telenursing promotes continuous monitoring, therapeutic adherence and clinical stability in patients with chronic diseases, promoting empowerment and self-management.
**Oncology pathways and symptom support**	Telecare helps manage the effects of cancer treatments, improves informational and emotional support, and ensures therapeutic continuity.
**Postoperative care and hospital-to-home transition**	Telenursing interventions facilitate the management of post-surgical symptoms, reduce complications and improve the safety of discharge.
**Telenursing in emergencies and disasters**	Telemedicine supports triage, clinical decision-making and resource coordination in critical contexts, ensuring timeliness and safety.
**Digital skills and nursing education**	Studies highlight the importance of developing digital, relational and communication skills to operate effectively in teleassistance.
**Economic and organizational impact**	Nursing telemedicine contributes to cost reduction, the appropriate use of resources, and the reorganization of care models.

## Data Availability

No new data were created or analyzed in this study. Data sharing is not applicable as this article is based exclusively on previously published literature.

## Supplementary Materials

The following supporting information can be downloaded at: https://www.mdpi.com/article/10.3390/nursrep16050161/s1, PRISMA 2020 Checklist. Reference [62] is cited in the supplementary materials.

## Public Involvement Statement

No public involvement in any aspect of this research.

## Guidelines and Standards Statement

This manuscript was developed in accordance with the Preferred Reporting Items for Systematic Reviews and Meta-Analyses extension for Scoping Reviews (PRISMA-ScR). The PRISMA 2020 Checklist see Supplementary Materials.

## Abbreviations

The following abbreviations are used in this manuscript:

PRISMA-ScR	Preferred Reporting Items for Systematic Reviews and Meta-Analyses Extension for Scoping Reviews
JBI	Joanna Briggs Institute
WHO	World Health Organization
ICT	Information and Communication Technologies
AI	Artificial Intelligence
EHR	Electronic Health Record
mHealth	Mobile Health
eHealth	Electronic Health
QoL	Quality of Life
